# Association of osteoporotic fractures of femoral neck and femoral neck geometric parameters in native Chinese women

**DOI:** 10.1186/s12891-024-07483-1

**Published:** 2024-05-03

**Authors:** Lin Li, Yi Shen, Li-Hua Tan, Hong Zhang, Ru-Chun Dai, Ling-Qing Yuan, Zhi-Feng Sheng, Xi-Yu Wu

**Affiliations:** 1 National Clinical Research Center for Metabolic Diseases, Hunan Provincial Key Laboratory of Metabolic Bone Diseases, Department of Metabolism and Endocrinology, The Second Xiangya, Hospital of Central South University, Changsha, 410011 China; 2 Department of Endocrinology and Metabolism, The Affiliated Changsha Hospital of Xiangya School of Medicine, Central South University, Changsha, China; 3 Department of Orthopedics, The Second Xiangya Hospital, Central South University, Changsha, 410011 China; 4 Department of Radiology, The Second Xiangya Hospital, Central South University, Changsha, 410011 China

**Keywords:** Femoral neck fracture, Femoral neck geometric parameters, Osteoporosis, Fracture risk

## Abstract

**Background:**

Although it is generally believed that the femoral neck fracture is related to the femoral neck geometric parameters (FNGPs), the association between the risk of osteoporotic fracture of the femoral neck and FNGPs in native Chinese women is still unclear.

**Methods:**

A total of 374 female patients (mean age 70.2 ± 9.32 years) with osteoporotic fracture of the femoral neck, and 374 non-fracture control groups were completely matched with the case group according to the age ratio of 1:1. Using DXA bone densitometer to measured eight FNGPs: the outer diameter (OD), cross-sectional area (CSA), cortical thickness (CT), endocortical diameter (ED), buckling ratio (BR), section modulus (SM), cross-sectional moment of inertia (CSMI), and compressive strength index (CSI) at the narrowest point of the femoral neck.

**Results:**

Compared with the control group, the average values of OD (2.9%), ED (4.5%), and BR (26.1%) in the patient group significantly increased (*p* = 0.015 to < 0.001), while CSA (‒15.3%), CT (‒18.2%), SM (‒10.3%), CSMI (‒6.4%), and CSI (‒10.8%) significantly decreased (all *p* < 0.001). The prevalence of osteoporosis in the lumbar spine, femoral neck, and total hip was, respectively, 82%, 81%, and 65% in fracture patients. Cox proportional hazard model analysis showed that in the age adjusted model, the fracture hazard ratio (HR) of CSA, CT, BR, SM, and CSI significantly increased (HRs = 1.60‒8.33; 95% CI = 1.08‒16.6; all *p* < 0.001). In the model adjusted for age and femoral neck BMD, HRs of CT (HRs = 3.90‒8.03; 95% CI = 2.45‒15.1; all *p* < 0.001) and BR (HRs = 1.62‒2.60; 95% CI = 1.20‒5.44; all *p* < 0.001) were still significantly increased.

**Conclusion:**

These results suggest that the majority of osteoporotic fractures of the femoral neck of native Chinese women occur in patients with osteoporosis. CT thinning or BR increase of FNGPs may be independent predictors of fragility fracture of femoral neck in native Chinese women unrelated to BMD.

## Background

Femoral neck fracture is the most serious type of osteoporotic fracture, accounting for approximately 49‒57% of hip fractures [1–3]. The one-year mortality rate associated with these fractures is approximately 14‒36% [4], up to 50% of patients with femoral neck fractures are permanently disabled or unable to return to their pre-fracture mobility [5]. The incidence of hip fracture in women is much higher than that in men [6–8], and it is a common disease that seriously endangers the health of old women. Epidemiological studies have shown that there are regional differences in the incidence of hip fractures, such as with lower incidence in Beijing than in the Hong Kong and Taiwan populations of China [9, 10], with the highest incidence in the Nordic population and the lowest incidence rates in Chinese mainland and Africans [11], with more than 10-fold differences in hip fracture risk and fracture probability between different countries [6]. Studies have shown that the incidence of hip fractures is declining in populations in North American countries, but it continues to increase in many Asian countries [9, 11]. Studies have also highlighted racial differences in the incidence of hip fractures, such as higher rates in white women than in black women in the United States [12, 13], lower rates in Canadians than in Americans and Germans [14].

The main cause of osteoporotic fractures of the femoral neck is a reduction in bone strength, which is primarily determined by bone mineral density (BMD) [15, 16] with other factors such as bone geometry, remodeling state and microstructure also playing important roles [16, 17]. The risk of femoral neck fracture is strongly associated with the hip geometric parameters such as hip axis length, femoral neck angle, and femoral neck width [18]. Hip axis length, femoral neck strength index, femoral neck length and cross-sectional area (CSA) are risk factors for female hip and femoral neck fractures independent of age and BMD [19, 20]. Thus, an assessment of the relationship between FNGPs and the risk of osteoporotic femoral neck fractures in different populations is important to improve the ability of predicting the risk of femoral neck fracture.

Although quantitative computed tomography (QCT) can obtain three-dimensional images and has advantages in measuring the true volume density and bone geometry of bone trabeculae or cortical bone, QCT is expensive in equipment and measurement costs, and slow in measurement, especially for subjects with large radiation doses [21, 22]. Compared with QCT technology, the equipment cost or measurement cost and measurement time of dual-energy X-ray absorptiometry (DXA), which is widely used in clinical practice, is only about one-fifth and one-tenth of that of QCT. In particular, the radiation dose of the hip measured by QCT is 2.5-3.0 mSv, while the radiation dose of DXA is only 0.009 mSv [21]. The radiation dose of QCT is about 280–330 times that of DXA [21, 22]. Therefore, DXA technology has the advantages of low cost, fast measurement speed and low radiation dose [21–23], and is highly correlated with QCT measurement results [24], so it has been widely used in clinical practice. We used DXA to measure FNGPs and studied the association between femur neck fragility fractures and FNGPs in Chinese local women.

## Methods

### Participants

Between March 2015 and October 2021, 374 patients with osteoporotic femoral neck fractures who met the inclusion criteria were identified, whose age was 42‒93 years (mean 70.2 ± 9.32 years). These patients with femoral neck fractures came to our orthopedics department for treatment, and after questioning and X-ray photos, they were diagnosed as osteoporotic femoral neck fractures. The inclusion criteria for osteoporotic fractures of the femoral neck were the presence of symptoms of a femoral neck fracture and admission to the hospital to report a femoral neck fracture that occurred with or without a fall from or below standing height. Femoral neck fractures were confirmed by a radiologist on proximal femoral radiographs, and BMD and FNGPs were measured using normal images on the non-fracture side of the patient’s proximal femur. Cases with only one femoral neck fracture were referred to as simple femoral neck fracture (SFNF), and those with a previous fragility fracture at another skeletal site were referred to as femoral neck fractures with other fractures (FNFOF). Patients were excluded if they had femoral neck fractures due to trauma such as a car accident or a fall from a chair and above, femoral neck fractures due to medication use or secondary osteoporosis, or bilateral hip fractures.

Data on 374 control individuals were obtained from the reference population of our previously established FNGPs reference database [25], and the control and case groups were fully matched by age in a 1:1 ratio. The inclusion criteria for individuals in the control group were no history of fractures, osteosclerosis, skeletal fluorosis, or abnormally increased BMD. The study was approved by the Ethics Committee of the Second Xiangya Hospital of Central South University, and informed consent was obtained from all participants. All participants were of Han ethnicity.

### BMD and FNGPs Measurement

Bone mineral content (BMC), projected bone area (BA), and BMD measurements of the lumbar spine (L1‒L4), femoral neck (FN), and total hip were obtained using DXA (Hologic Delphi A; Hologic, Bedford, MA, USA). For patients who had undergone a hip fracture or hip replacement, measurements were obtained for the contralateral proximal femur. Hip measurements obtained from patients with bilateral hip fractures were discarded, and the patients with these fractures were excluded. Cases in which the lumbar spine was filled with artificial bone cement or fitted with metal brackets were excluded from imaging analysis of the lumbar spine. BMD was measured twice with DXA bone densitometry in 33 participants, and the root-mean-square coefficient of variation (RMSCV) for the lumbar spine, femoral neck, and total hip was 0.86%, 1.17%, and 0.88%, respectively. The long-term (> 17 years) variation coefficient of the daily quality control phantom measured by DXA was < 0.45%. The sex-specific BMD T-scores of the lumbar spine, femoral neck, and total hip were calculated using the BMD reference database established in our laboratory [26], which was defined by the World Health Organization (WHO) [27] and compared with the peak BMD for the same sex: participants with BMD T-score > ‒1.0 were considered to have normal BMD, while those with T-scores ≤ ‒1.0 to > ‒2.5 and ≤ ‒2.5 were considered to show low bone mass and osteoporosis, respectively.

The femoral neck BA, BMC, and BMD were measured by DXA, and FNGP was calculated using the reported Eqs. [28, 29]. We measured a total of eight FNGPs, namely, outer diameter (OD), cross-sectional area (CSA), cortical thickness (CT), endocortical diameter (ED), buckling ratio (BR), section modulus (SM), cross-sectional moment of inertia (CSMI), and compression strength index (CSI) at the narrowest point of the femoral neck. OD is the femoral neck outer diameter at the middle point of the femoral neck axis length, CSA is an indicator of bone axial strength, CT is an estimate of mean cortical thickness, ED is the endocortical diameter of the femoral neck, BR is an index of bone structural instability, SM is an index of bone bending strength indicating the bending resistance of a tube, CSMI is an index of bone stiffness, and CSI is a composite index of resistance to the pressure of the main shaft of the femoral neck (CSI = BMD × OD/body weight [29]). Using a case-control study approach, we studied geometric parameters at the narrowest point of the femoral neck in patients with osteoporotic fractures of the femoral neck and controls who were fully matched for age.

### Statistical analysis

Data were analyzed and plotted using SPSS V23.0 for Windows Software (SPSS Inc., Chicago, IL, USA). A one-sample Kolmogorov-Smirnov test (K-S test) was used to investigate whether the data were normally distributed. The K-S test showed that the age, height, weight, body mass index (BMI), BMD, and FNGPs of the participants showed a normal distribution (all Z = 0.629‒1.276; all *p* = 0.824‒0.077). Therefore, the mean and standard deviation were used to express these parameters in the case group, control group, and fracture subgroups. One-way analysis of variance (ANOVA) was used to determine significant differences in the mean values among the groups. Chi-square test was used to compare the percentage of osteoporosis, osteopenia, or normal BMD in fracture groups. FNGPs were stratified by tertiles, and the Cox proportional hazards model and multivariate analysis were used to evaluate the association of these parameters with the risk of osteoporotic fracture of the femoral neck by evaluating fracture hazard ratios (HR) and 95% confidence intervals (95% CI). Multivariable analysis was performed using two models, one adjusted for age, another adjusted for age and femoral neck BMD. Statistical significance was defined by *p* < 0.05.

## Results

### Characteristics of participants

The mean body weight, BMI, and BMD at various skeletal sites in the case group were significantly lower than those in the control group (all *p* = 0.007 to < 0.001) (Table 1). In comparison with the control group, the case group showed significantly higher mean OD (2.9%), ED (4.5%), and BR (26.1%) (all *p* = 0.015 to < 0.001) and significantly lower CSA (‒15.3%), CT (‒18.2%), SM (‒10.3%), CSMI (‒6.4%), and CSI (‒10.8%) (all *p* < 0.001). In the fracture subgroups, the mean age of the FNFOF group was significantly higher than that of the SFNF group, and the mean height, LS-BMD, and hip-BMD of the FNFOF group were significantly lower than those of the SFNF group. The prevalence of osteoporosis in the lumbar spine, femoral neck, and total hip of patients with femoral neck fractures was 82%, 81%, and 65%, respectively, and the corresponding percentages in the control group were 47%, 37%, and 30%, respectively; the prevalence in the case group was significantly higher than that in the control group at all sites (all *p* < 0.001). The prevalence of osteopenia in these skeletal sites was, respectively, 16%, 18%, and 32% in the case group, and 41%, 47%, and 52% in the control group; the values in the control group were significantly higher than those in the case group (all *p* < 0.001). The rates of normal BMD in the lumbar spine, femoral neck, and total hip were, respectively, 2%, 1%, and 3% in the case group and 12%, 16%, and 18% in the control group; the values in the control group were significantly higher than those in the case group (all *p* < 0.001).

**Table 1:** Comparison of basic characteristics among cases of fractures and controls

Parameter	Control	Case	Fracture subgroup	
			SFNF	FNFOF
n (%)	374	374	200 (53.5)	174 (46.5)
Age (years)	70.2 ± 9.32	70.2 ± 9.32	69.0 ± 9.64	71.6 ± 8.77^c^
Height (cm)	151.7 ± 5.37	152.0 ± 6.71	153.5 ± 5.95^b^	150.3 ± 7.11^bc^
Weight (kg)	54.8 ± 8.99	52.2 ± 7.84^a^	52.6 ± 8.04	51.7 ± 7.60
BMI (kg/m^2^)	23.8 ± 3.59	22.6 ± 3.07^a^	22.3 ± 3.12	22.9 ± 2.99
LS-BMD (g/cm^2^)	0.754 ± 0.144	0.662 ± 0.115^a^	0.684 ± 0.116^b^	0.637 ± 0.108^bc^
FN-BMD (g/cm^2^)	0.601 ± 0.112	0.494 ± 0.090^a^	0.493 ± 0.098	0.495 ± 0.080
Hip-BMD (g/cm^2^)	0.672 ± 0.132	0.573 ± 0.109^a^	0.587 ± 0.116	0.557 ± 0.097^c^
OD (cm)	3.09 ± 0.23	3.18 ± 0.32^a^	3.16 ± 0.32	3.21 ± 0.32
CSA (cm^2^)	1.77 ± 0.35	1.50 ± 0.33^a^	1.48 ± 0.35	1.51 ± 0.29
ED (cm)	2.87 ± 0.24	3.00 ± 0.32^a^	2.97 ± 0.32	3.03 ± 0.32
CT (mm)	11.4 ± 2.23	9.32 ± 1.74^a^	9.25 ± 1.90	9.28 ± 1.54
BR	14.2 ± 3.61	17.9 ± 4.59^a^	17.9 ± 5.00	17.9 ± 4.08
SM (cm^3^)	0.998 ± 0.222	0.895 ± 0.261^a^	0.879 ± 0.276	0.912 ± 0.242
CSMI (cm^4^)	1.56 ± 0.43	1.46 ± 0.58^a^	1.42 ± 0.60	1.50 ± 0.54
CSI (g/kg × m)	3.42 ± 0.58	3.05 ± 0.67^a^	2.96 ± 0.67	3.11 ± 0.66

Values are mean ± SD. ^a^*p*=0.020 to < 0.001 compared with control; ^b^*p*=0.048 to < 0.001 compared with case; ^c^*p*=0.040 to < 0.001 compared with SFNF. BMI: body mass index; LS: lumbar spine; BMD: bone mineral density; FN: femoral neck; Hip: total hip; OD: outer diameter; CSA: cross-sectional area; CT: cortical thickness; ED: endocortical diameter; BR: buckling ratio; SM: section modulus; CSMI: cross-sectional moment of inertia; CSI: compression strength index; SFNF: simple FN fracture; FNFOF: FN fracture with other fracture

### Distribution trend of FNGPs

**Fig. 1:**
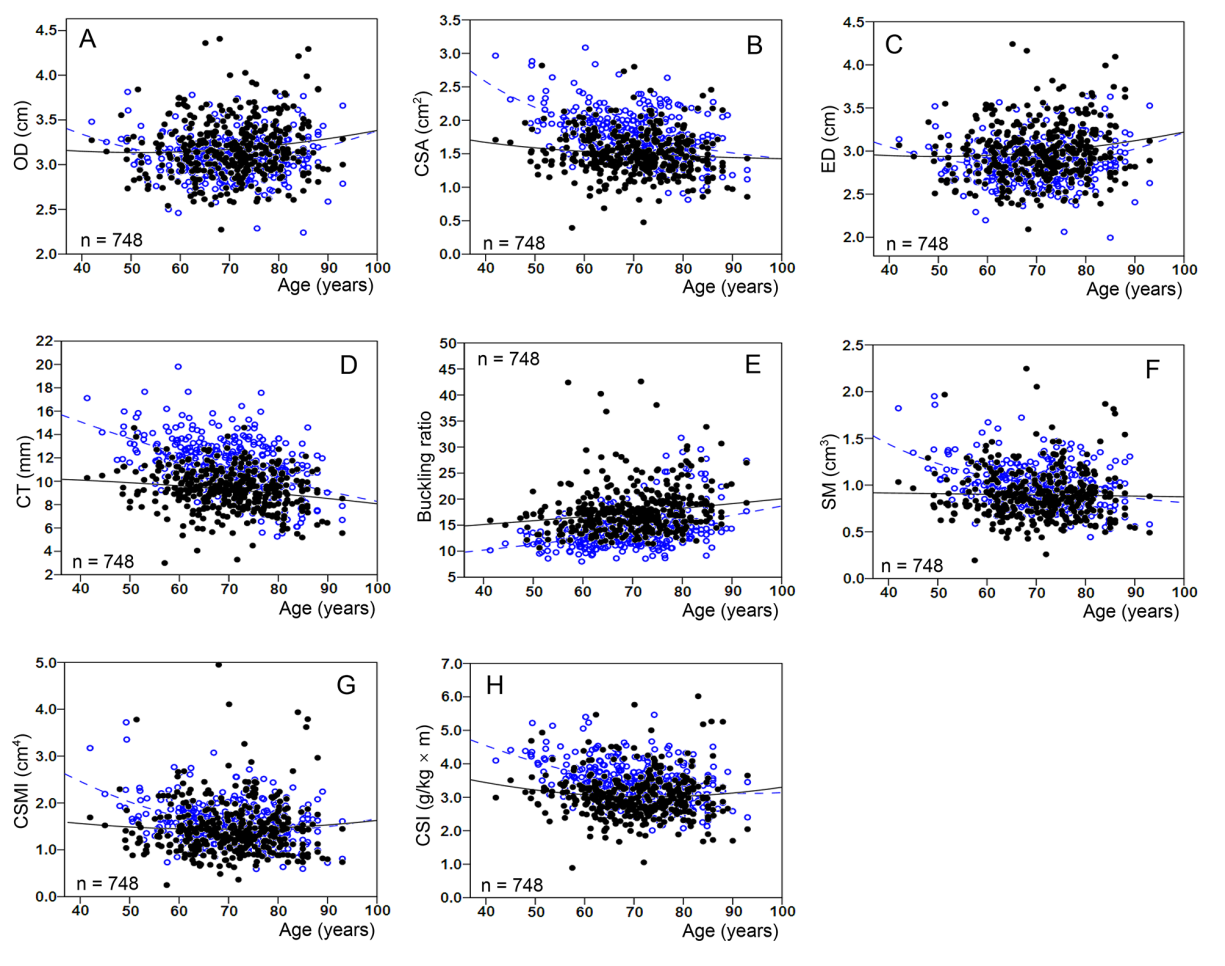
Distribution trends of FNGPs in female femoral neck fracture patients (filled dots and full line) and controls (open dots and dotted line). FNGPs: femoral neck geometric parameters; OD: outer diameter; CSA: cross-sectional area; ED: endocortical diameter; CT: cortical thickness; SM: section modulus; CSMI: cross-sectional moment of inertia; CSI: compression strength index

Figure 1 shows the distribution trend of FNGPs in the neck of femur fracture group and the control group. According to the scatter plot, the scatter points of OD (Fig. 1A), ED (Fig. 1C), SM (Fig. 1F), and CSMI (Fig. 1G) in the case and control groups almost showed a staggered distribution trend. Most of the scatter points of CSA (Fig. 1B), CT (Fig. 1D), and CSI (Fig. 1H) in the case group appeared to be at lower levels, while most of these scatter points in the control group were at higher levels. In contrast, the scatter points of the geometric parameter BR in the case group were mostly at a higher level and those in the control group were mostly at a lower level (Fig. 1E).

### Fracture hazard ratios

CSA, CT, SM, CSMI, and CSI of participants were stratified in descending order (T1 was the highest, T3 was the lowest), while OD, ED, and BR were stratified in ascending order of tertiles (T1 was the lowest, T3 was the highest), and multivariate Cox proportional hazards model analysis (Table 2) showed that with the first tertile group (T1) as the reference value in the age-adjusted model, changes in the FNGPs CSA, CT, BR, SM, CSMI, and CSI resulted in increases in HR1. The HR1 ranged from 1.60 to 8.33 (all *p* < 0.001). In the model adjusted by age and neck of femur BMD, the HR2 of CT and BR still increased significantly, and their range was 1.62‒8.03 (all *p* < 0.001).

**Table 2:** The effect of femoral neck geometric parameters stratification on fracture hazard ratio (HR)

Variable	HR model 1 (HR1; 95% CI)				HR model 2 (HR2; 95% CI)		
	T1 group	T2 group	T3 group		T1 group	T2 group	T3 group
OD	Ref	0.83 (0.54–1.27)	**1.34 (1.07–1.68)**		Ref	0.75 (0.37–1.49)	1.20 (0.91–1.59)
CSA	Ref	**3.71 (2.17–6.33)**	**2.94 (2.09–1.46)**		Ref	0.87 (0.40–1.87)	0.90 (0.48–1.70)
ED	Ref	1.24 (0.79–1.94)	**1.62 (1.27–2.05)**		Ref	1.01 (0.48–2.13)	1.26 (0.95–1.68)
CT	Ref	**8.33 (4.17–16.6)**	**3.94 (2.61–5.95)**		Ref	**8.03 (3.97–15.1)**	**3.90 (2.45–5.45)**
BR	Ref	**6.64 (3.52–12.5)**	**3.98 (2.63–6.01)**		Ref	**2.60 (1.25–5.44)**	**1.62 (1.20–3.19)**
SM	Ref	**1.65 (1.08–2.52)**	**1.93 (1.47–2.52)**		Ref	0.89 (0.49–1.59)	0.79 (0.51–1.22)
CSMI	Ref	1.20 (0.81–1.78)	**1.60 (1.25–2.06)**		Ref	0.63 (0.35–1.10)	0.93 (0.66–1.32)
CSI	Ref	**2.35 (1.48–3.71)**	**2.10 (1.62–2.71)**		Ref	1.54 (0.90–2.65)	0.95 (0.65–1.39)

Model 1 is adjusted for the age of traditional fracture risk; Model 2 is adjusted for the age and neck of femur BMD of traditional fracture risks. CSA, CT, SM, CSMI and CSI respectively by tertile descending stratification; OD, ED and BR respectively by tertile ascending stratification. Significant HRs are shown in bold (all *p* < 0.001). OD: outer diameter; CSA: cross-sectional area; ED: endocortical diameter; CT: cortical thickness; BR: buckling ratio; SM: section modulus; CSMI: cross-sectional moment of inertia; CSI: compression strength index; T1: first tertile; T2: second tertile; T3: third tertile

## Discussion

Our study showed that the rate of osteoporosis in the lumbar spine, femoral neck or total hip of these patients with femoral neck fractures was 65‒82%, and the rate of low bone mass and normal BMD was 18‒35%. Other studies have shown that the rate of osteoporosis in women with fragility fractures is only 18‒40%, and the rate of low bone mass and normal BMD is 60‒82% [30–32]. Among female hip fracture patients, the rate of hip osteoporosis accounted for 46%, and the rate of low bone mass and normal BMD reached 54% [33]. These significant differences in the results may be attributable to racial differences and the different fracture sites in the study populations. The present study also showed that fragility fractures of the femoral neck also occurred in adults younger than 50 years of age, with approximately 2.4% of patients ≤ 50 years of age. Other studies have showed that among all patients with hip fractures, approximately 2‒11% of them are younger than 50 years old [34]. The latest research shows that the proportion of patients with hip fractures under the age of 50 with femoral neck fractures is 58% [35].

This study used Leslie et al. [19] to calculate the femoral neck fracture risk ratio (HR) by adjusting for age and adjusting for age and femoral neck BMD models. In the age adjusted model (HR1), FNGP was grouped according to the tertiles, with the first group (T1) as the reference value, CSA, CT, BR, SM, and CSI showed a significant 1.65‒8.33-fold increase in fracture risk (HR1) in the T2 and T3 groups. For OD, ED, and CSMI, the fracture risk did not significantly increase in the T2 group, but only in the T3 group, the fracture risk (HR1) significantly increased by 1.34‒1.62 times. These findings suggest that, after controlling for the effect of age, almost all these FNGPs are associated with an increased risk of femoral neck fracture, with changes in CT and BR levels leading to the highest risk of femoral neck fracture. As CT levels decrease, the fracture risk in the T2 and T3 groups increases by 8.33 and 3.94 times, respectively; as BR levels increase, the fracture risk of these two groups increases by 6.64 times and 3.98 times, respectively. In the model adjusted for age and femoral neck BMD (HR2), the risk of femoral neck fracture still significantly increased as CT levels decreased and BR levels increased. The fracture risk ratios of CT level changes in the two models (HR1 and HR2) were similar (Table 2), indicating that changes in femoral neck BMD had a smaller impact on CT but a greater impact on BR, as in the HR1 model, the risk ratios of the BR T2 and T3 groups were 6.64 and 3.98, respectively, in the HR2 model, this risk ratio was reduced to 2.60 and 1.62, respectively. These findings suggest that CT and BR may be independent predictors of femoral neck fracture risk independent of age and femoral neck BMD. Another study showed that the geometric parameters CSA and OD of the femoral neck are independent risk factors for femoral neck fractures in Korean women [20], which is different from our research results. This suggests that there may be racial differences in the association between FNGPs and the risk of femoral neck fractures, or it may be related to the different design methods of Han et al. [20] and the small sample size of femoral neck fractures (*n* = 84). Iolascon et al. [36] showed that hip axis length (HAL) was longer and all geometric parameters were poorer in women with hip fracture, suggesting that hip structure analysis (HSA) has an impact on the risk of hip fracture in postmenopausal women. It can provide additional information on the spatial distribution of bone mass, which is closely related to bone strength. Other studies have reported that the rapid bone turnover of women in menopause accelerates bone resorption on the endoosseous surface, leading to CT thinning of the femoral neck and reducing the stability of bone structure [37]. Cortical thinning causes a reduction in the CSA and SM of the femoral neck, and decreases the ability of bone to resist axial stress and bending stress [38]. The changes in these parameters are important risk factors for femoral neck fragility fracture, which can explain the higher incidence of femoral neck fracture in the elderly. There are also studies indicating that, in comparison with patients with femoral neck fractures, thinner femoral shaft cortical bone is more common in greater trochanter fractures [39]. The femoral shaft cortical thickness index is negatively correlated with the risk of death caused by hip fracture, and the smaller the cortical thickness index, the greater the risk of death [40]. Recent studies have shown an increase in risk factors for fragility fractures during the COVID-19 pandemic [41], such as rapid muscle atrophy due to prolonged immobility, vitamin D deficiency and widespread use of corticosteroids that accelerate bone loss and thus increase the risk of fractures associated with falls.

The limitation of this study, as described by other studies [42, 43], is that the accuracy of DXA in describing bone geometric features is inherently limited, and the deduced 3D model of femoral neck cross section may be different from the real bone geometry of individual participants. However, the geometric features of femoral neck described by two-dimensional data derived from DXA have been proven to be highly correlated with three-dimensional QCT data [44]. Second, the assumption that the CT of the femoral neck cross section is a uniform round annular cortical shell is not completely consistent with the actual situation, which may affect the accuracy of this parameter. Third, the differences in soft tissue thickness around the proximal femur of the participants may affect the projected bone image and estimated FNGPs obtained by DXA scanning.

## Conclusion

In the model adjusted for age and femoral neck BMD, CT decline or BR increase were independent risk factors for femoral neck fragility fracture, and the risk of femoral neck fracture decreased linearly with a reduction in CT and an increase in BR. Understanding the relationship between these parameters and the risk of femoral neck fracture may have important reference value for fracture risk assessment and fracture prevention.

## Data Availability

Data is provided within the manuscript.

## Abbreviations

FNGPs: Femoral neck geometric parameters
QCT: Quantitative computed tomography
DXA: Dual-energy X-ray absorptiometry
OD: Outer diameter
CSA: Cross-sectional area
CT: Cortical thickness
ED: Endocortical diameter
BR: Buckling ratio
SM: Section modulus
CSMI: Cross-sectional moment of inertia
CSI: Compression strength index
CI: Confidence intervals
BMD: Bone mineral density
SFNF: Simple FN fracture
FNFOF: Femoral neck fracture with other fracture
BMC: Bone mineral content
BA: Bone area
FN: Femoral neck
RMSCV: Root-mean-square coefficient of variation
BMI: Body mass index
LS: Lumbar spine
